# Geometrical illusions are not always where you think they are: a review of some classical and less classical illusions, and ways to describe them

**DOI:** 10.3389/fnhum.2014.00856

**Published:** 2014-10-28

**Authors:** Jacques Ninio

**Affiliations:** Laboratoire de Physique Statistique, Physics Department, Ecole Normale Supérieure/PSL Research UniversityParis, France

**Keywords:** geometrical illusions, classification, measurement theories, convexity, orthogonal expansion

## Abstract

Geometrical illusions are known through a small core of classical illusions that were discovered in the second half of the nineteenth century. Most experimental studies and most theoretical discussions revolve around this core of illusions, as though all other illusions were obvious variants of these. Yet, many illusions, mostly described by German authors at the same time or at the beginning of the twentieth century have been forgotten and are awaiting their rehabilitation. Recently, several new illusions were discovered, mainly by Italian authors, and they do not seem to take place into any current classification. Among the principles that are invoked to explain the illusions, there are principles relating to the metric aspects (contrast, assimilation, shrinkage, expansion, attraction of parallels) principles relating to orientations (regression to right angles, orthogonal expansion) or, more recently, to gestalt effects. Here, metric effects are discussed within a measurement framework, in which the geometric illusions are the outcome of a measurement process. There would be a main “convexity” bias in the measures: the measured value m(x) of an extant x would grow more than proportionally with x. This convexity principle, completed by a principle of compromise for conflicting measures can replace, for a large number of patterns, both the assimilation and the contrast effects. We know from evolutionary theory that the most pertinent classification criteria may not be the most salient ones (e.g., a dolphin is not a fish). In order to obtain an objective classification of illusions, I initiated with Kevin O'Regan systematic work on “orientation profiles” (describing how the strength of an illusion varies with its orientation in the plane). We showed first that the Zöllner illusion already exists at the level of single stacks, and that it does not amount to a rotation of the stacks. Later work suggested that it is best described by an “orthogonal expansion”—an expansion of the stacks applied orthogonally to the oblique segments of the stacks, generating an apparent rotation effect. We showed that the Poggendorff illusion was mainly a misangulation effect. We explained the hierarchy of the illusion magnitudes found among variants of the Poggendorff illusion by the existence of control devices that counteract the loss of parallelism or the loss of collinearity produced by the biased measurements. I then studied the trapezium illusion. The oblique sides, but not the bases, were essential to the trapezium illusion, suggesting the existence of a common component between the trapezium and the Zöllner illusion. Unexpectedly, the trapeziums sometimes appeared as twisted surfaces in 3d. It also appeared impossible, using a nulling procedure, to make all corresponding sides of two trapeziums simultaneously equal. The square-diamond illusion is usually presented with one apex of the diamond pointing toward the square. I found that when the figures were displayed more symmetrically, the illusion was significantly reduced. Furthermore, it is surpassed, for all subjects, by an illusion that goes in the opposite direction, in which the diagonal of a small diamond is underestimated with respect to the side of a larger square. In general, the experimental work generated many unexpected results. Each illusory stimulus was compared to a number of control variants, and often, I measured larger distortions in a variant than in the standard stimulus. In the Discussion, I will stress what I think are the main ordering principle in the metric and the orientation domains for illusory patterns. The convexity bias principle and the orthogonal expansion principles help to establish unsuspected links between apparently unrelated stimuli, and reduce their apparently extreme heterogeneity. However, a number of illusions (e.g., those of the twisted cord family, or the Poggendorff illusions) remain unpredicted by the above principles. Finally, I will develop the idea that the brain is constructing several representations, and the one that is commonly used for the purpose of shape perception generates distortions inasmuch as it must satisfy a number of conflicting constraints, such as the constraint of producing a stable shape despite the changing perspectives produced by eye movements.

## Introduction

The field of geometrical illusions is in a kind of unhealthy Babel tower situation. Year after year, both expert authors, and new-comers in the field propose all-embracing theories of geometric illusions (for instance: Bulatov et al., 1997; Prinzmetal and Beck, 2001; Purves et al., 2001; Changizi and Widders, 2002; Fermuller and Malm, 2004; Nemati, 2009; Day, 2010). And there are countless theoretical articles on particular illusions, the favorite ones being the Müller-Lyer and the Poggendorff illusions. Large collections of illusory figures can be found in various web sites (e.g., Akiyoshi Kitaoka's “Colloquium of visual illusions” and Michael Bach's site on “Optical illusions & visual phenomena”) and in specialized books (e.g., Luckiesh, 1922; Tolanski, 1964; Robinson, 1972; Coren and Girgus, 1978; Wade, 1982; Da Pos and Zambianchi, 1996; Vicario, 2011). The last two books are particularly precise about historical sources for the illusions, and many of the references in this review are borrowed from these books.

As stated by Ninio and Pinna (2006), “illusions, even when they are given different names, often seem to have strong family ties and form a continuum.” Various authors attempt to put some order, and regroup the illusions into classes, for instance metric vs. orientation illusions, illusions of contrast vs. assimilation, of shrinkage vs. expansion, illusions due to eye-movements, spatial filtering, depth processing, or local vs. gestalt effects.

Authors of philosophical books, or of popular science books on visual paradoxes do not hesitate to offer their own explanations of illusions, without any guilty conscience about their ignorance of experimental results, and without an awareness of the already published refutations of their proposals. For instance, the vertical-horizontal illusion (a vertical segment appears longer than a horizontal segment of the same length) is often illustrated with an inverted letter T, of which the vertical branch seems longer than the horizontal one. Yet, it has been known for quite long that the apparently shorter size of the horizontal branch in the letter T is due to its being bisected by the vertical branch (Künnapas, 1955). There are also cases in which a certain effect is described, and I see just the opposite effect. This is not a drama for science, it merely implies that people may have different sensitivities to different effects, and when the illusion in a visual pattern has several components that work in different directions, the resulting overall effect may go in one direction for some people, and the opposite direction for others.

Respected scientists are often victims of their too narrow selection of stimuli. For instance Moulden and Renshaw (1979) discussed a visual effect in which the steps of a black staircase seem to form acute instead of right angles. They interpreted the effect as due to irradiation—the white background would nibble the black stair. However, Kitaoka (1998) ruined the theory by pointing out that the effect is maintained when black and white are interchanged. I can cite a number of articles on illusions that are immediately refutable by a counter-example, and a number of mathematical treatments in other fields that contain obvious mathematical mistakes in the derivations of the equations.

An external observer would find it extremely difficult to determine what has really been established experimentally over the last 50 years, on what issues there is a theoretical consensus, what are the unsettled issues, and what kind of data are sorely needed, etc. Controversies are respectable facets of scientific activities. Unfortunately, in the field of geometrical illusions, many theoretical articles pay little attention to the experimental results, or to the extreme diversity of known illusions or their variants.

In this review, I will first present the corpus of some known and some less-known but equally legitimate illusions. I will then examine the proposition according to which the perceived shape of a figure may be related to the original figure through a kind of cartographic transformation: measurements would be taken on the figure to be perceived, then combined and adjusted, and a representation would be produced. The representation may disagree, in some of its geometrical aspects from what we know of the initial figure, and we may then state that there is an illusion. I will present several principles that may be invoked in a cartographic frameworks, in particular a “convexity rule” for metric measurements, and an “orthogonal expansion rule” for orientation biases related to the Zöllner illusion. I will show that representation theories can solve apparent contradictions between some illusions and their apparent counter-illusions. On the other hand, I will show that there are some inescapable problems in shape perception that make it sometimes difficult to state whether a figure contains an illusion, or merely illustrates a legitimate paradox of shape perception.

After discussing measurement theories, I discuss the possibility of an objective classification of illusions, and present “orientation profiles” as a way to investigate objectively the relationships between illusions. I present published results on orientation profiles, and in particular published orientation profiles from Ninio and O'Regan (1999), in Poggendorf illusions and related patterns. Finally, I present in the Discussion some of my current best guesses on the geometric principles underlying various geometric illusions.

## The corpus of geometrical illusions

**Figure 1:**
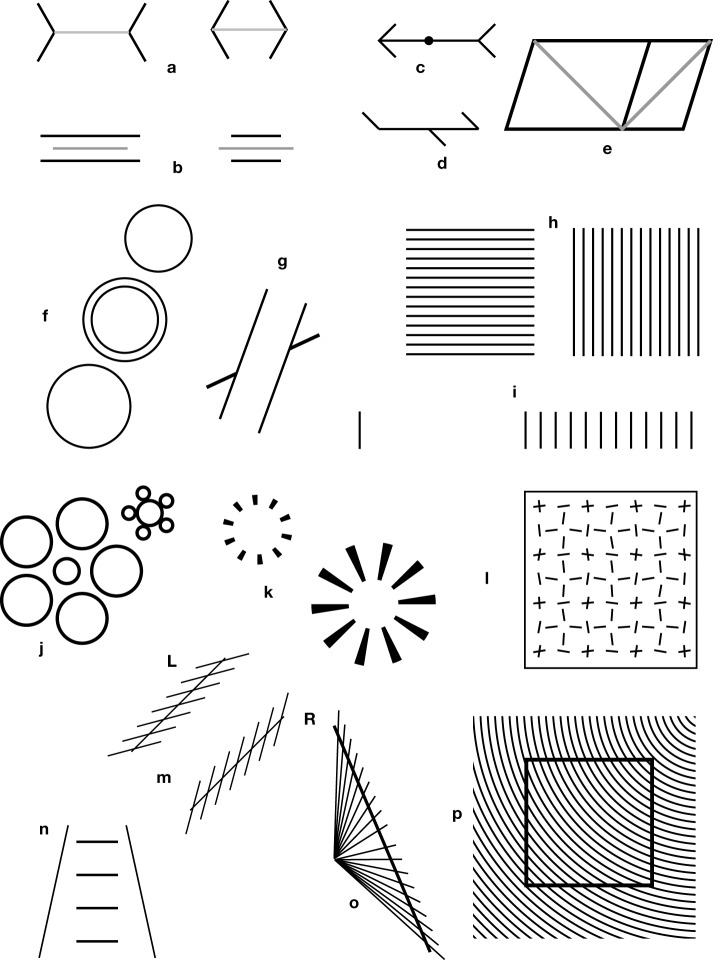
**Core illusions**. **(a)** A Müller-Lyer illusion, in its most popular form. The two gray segments have equal legths. **(b)** A variant of the Müller-Lyer illusion, proposed by Müller-Lyer himself, showing that contrary to popular belief, the fins are not necessary for the illusion. **(c)** A variation on the theme of ingoing and outgoing fins, proposed by Judd (1899). The dot is bisecting exactly the shaft, but appears displaced toward the ingoing fins. **(d)** A variation by Pegrassi (1904) on the previous Judd illusion. **(e)** Sander's parallelogram (1926). The diagonal on the left side seems longer than the diagonal on the right. It seems that the illusion can already be demonstrated with two triangles (**Figure 5h**). **(f)** Delboeuf illusion with circles. In the central pair of circles, the internal small circle is equal to the small external circle, but appears larger, and the surrounding large circle is equal to the external large circle, but seems smaller. **(g)** Poggendorff illusion. The two thick oblique segments are collinear, but appear to be misaligned. **(h)** Helmholtz' square illusion. The squares are equal, but are perceived as rectangles that are elongated in the direction that is orthogonal to the dividing segments. **(i)** Oppel-Kundt illusion: The subdivided half of the figure appears to be longer than the non-subdivided half. **(j)** Ebbinghaus or Titchener's illusion (Ebbinghaus, 1902). The two central circles are equal, but the one surrounded by small circles seems to be larger than the other. The illusory effect does not require circular geometry. It already works with triples of aligned elements. **(k)** A variation on this theme (from Ninio, 1998), designed as a hybrid between **(j)** and **(n)** or Figure 2f. **(l)** Variation on the bisection illlusion. The small isolated segments are equal in length to the segments that are associated in crosses, but appear to be longer (from Ninio, 1998). **(m)** Zöllner illusion. The two stacks are parallel, but seem to diverge at their tops (Zöllner, 1862). The “left-handed” and “right-handed” variants of the stacks, are labeled “L” and “R” according to the convention in Ninio and O'Regan (1996). **(n)** The so-called Ponzo illusion. The horizontal bars seem to increase in length, from bottom to top. **(o)** Hering illusion. The long line crossing the bundle is straight, but seems to be curved. **(p)** Variation on a theme by Ehrenstein (1925) and Orbison (1939): The black square seems to be trapezoidal, due to its intersections with the circles.

Most theoretical discussions revolve around a very small number of “core” geometrical illusions, ignoring most of the others. Some of these illusions are shown in Figure 1 or dispersed in other figures, as required by the discussed topics. These illusions are usually named according to their presumptive author (Zöllner, Poggendorff, Müller-Lyer, Delboeuf, Ebbinghaus, Helmholtz, Hering)—neglecting the facts that several authors invented a large number of illusory patterns, and that attribution errors are quite common in the field (see Da Pos and Zambianchi, 1996; Vicario, 2011 for historical details). There is the hidden assumption that other illusions are obvious variants of the core illusions.

**Figure 2:**
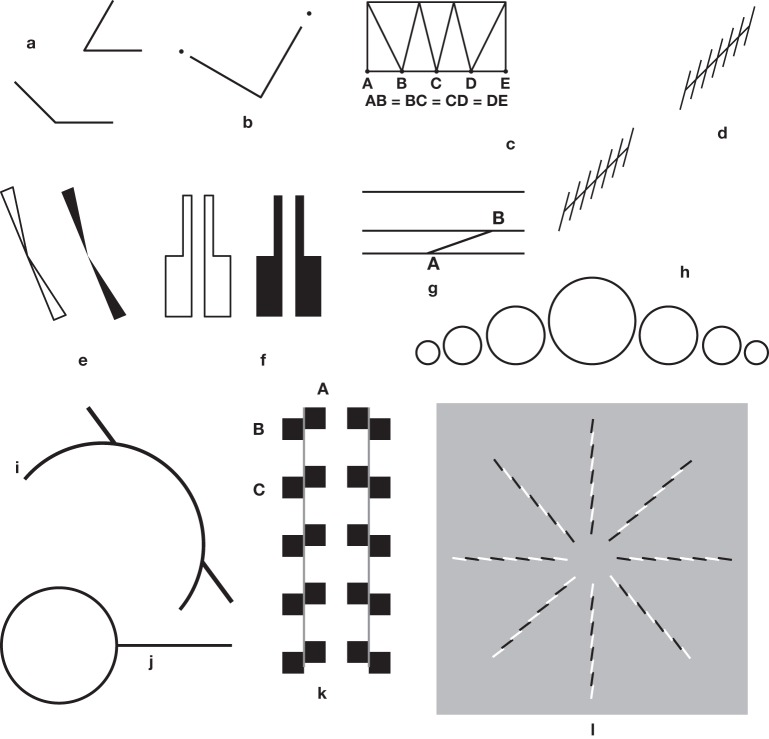
**Other old illusions**. **(a)** Again, a major Müller-Lyer illusion. The obtuse and the acute angles have arms of equal lengths, but those of the obtuse angle seem to be longer. **(b)** An illusion by Laska (1890). The two arms of the right angle are equal, but the one on the left, pointing toward the close dot seems shorter. **(c)** Dr Fee's 1888 illusion, reproduced in Ninio (1998). The bottom bases of the four triangles are equal, but the two central ones seem to be shorter than the others. **(d)** A variation by Botti (1909) on the Zöllner illusion. The axes of the two stacks are collinear, but seem to be misaligned. **(e)** An illusion by Bourdon (1902). The bases of the black or the white triangles are aligned, but the triangles seem to be slightly folded inward. **(f)** An illusion by Schumann (1900). The white or the black blocks seem to diverge at their top. **(g)** An illusion by Delboeuf (1865). The three long lines are parallel, but the top one seems to diverge from the two bottom ones. **(h)** An illusion by Lipps (1897) The circles have a common horizontal tangent, but seem to rest on a curved line making the central circles appear slightly higher than the lateral ones. **(i)** An illusion by Judd (1899)—an ancestor of the corner-Poggendorff illusion (Greene, 1988) shown in **Figure 10g**? **(j)** An illusory figure by Titchener (1901). The external segment seems to be larger than the diameter of the circle, yet they are equal. **(k)** The Münsterberg (1897) pattern, possibly the ancestor of a large number of illusions, including “checkered patterns,” and “café-wall” illusions. Superficially, the long lines together with the pairs of squares like AB form Zöllner stacks. However, it seems that the characteristic effects in the Münsterberg family are due to A–C patterns. **(l)** A variant of the twisted cord illusion, which belongs to the family of Fraser's illusions (Fraser, 1908). Here, the cords are collinear two by two, but seem to be misaligned, with the exception of the horizontal pair (from Ninio and Pinna, 2006).

Other illusions (Figure 2), often from German authors of the nineteenth century, are sometimes shown in books (e.g., Da Pos and Zambianchi, 1996; Vicario, 2011). Some of them remain as curiosities for specialists and have not yet acquired the status of major illusions.

**Figure 3:**
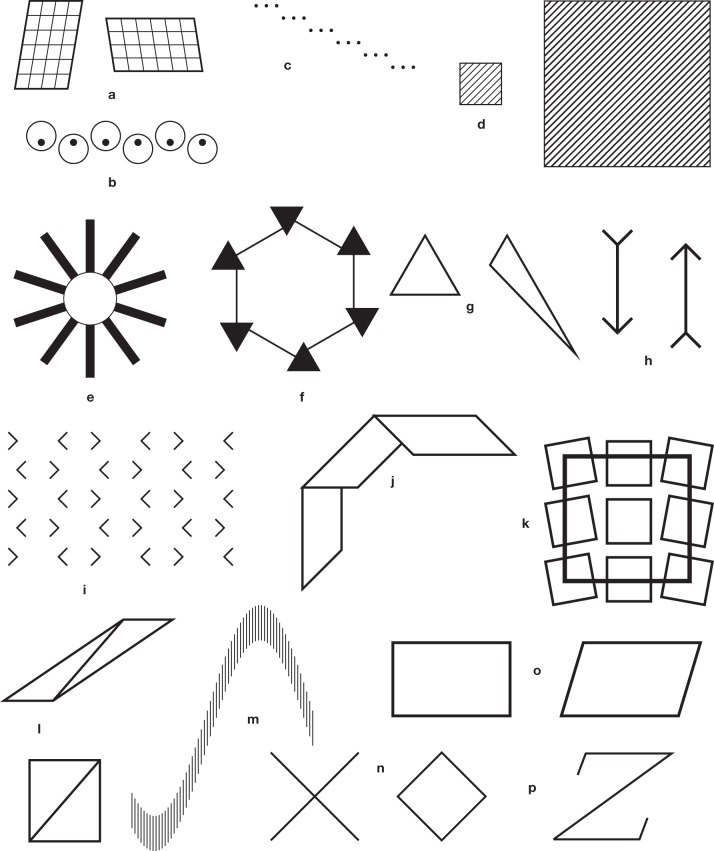
**Some recently discovered illusions**. **(a)** Shepard's tables (simplified). **(b)** Giovanelli's illusion (Giovanelli, 1966). The strict alignment of the black disks is not obvious. **(c)** Vicario's sloping steps. The dot triplets are all horizontal, but they seem to diverge (Vicario, 1978). **(d)** Vicario's rarefaction illusion (Vicario, 1972). The oblique lines have the same spacing in the left and the right squares, but those in the small square appear to have a wider separation. **(e)** The misangulation effect of Pinna (1991). The circle takes on a polygonal appearance. **(f)** An illusion by Gerbino (1978). The segments belong to the sides of a perfect hexagon, but seem to be displaced. **(g)** The triangle illusion of Kennedy et al. (2008). The two angles at the top of the triangles are equal, but the one belonging to the equilateral triangle seems to be larger than the other. A tentative explanation is provided in the legend to **Figure 5g**. **(h)** The receding arrow illusion (Holding, 1970). The arrow on the right seems to be shifted downward with respect to the arrow on the left. **(i)** Morinaga's paradox (Morinaga and Ikeda, 1965). The horizontal spacings of the “arrowheads” are perceived as predicted by the Müller-Lyer illusion of Figure 1a. On the other hand, the apices of the angles lie on vertical lines, but they appear shifted laterally in contradiction with the apparent horizontal spacings. **(j)** The illusion of Bressanelli and Massironi (2007). The three quadrangles have equal widths, but their width seems to increase from top to bottom. **(k)** Another illusion by Pinna (from Ninio and Pinna, 2006). The large square is perceived as a trapezium. **(l)** An illusion by Rausch (1952). The two diagonals are parallel, but the top one, in the parallelogram, seems to be closer to the vertical direction. **(m)** The sine illusion of Day and Stecher (1991). All the segments composing the pattern have equal lengths. This is a striking variation on a theme by Botti (1906). **(n)** Another illusion by Day (2006). The half segments belonging to the cross are equal to the sides of the diamond, but appear to be longer. **(o)** Another illusion by Rausch (1952). The rectangle and the parallelogram have equal long sides, and equal heights, yet the parallelogram seems to be larger than the rectangle. **(p)** The “Z” illusion (Fischer, 1999). The upper or lower short segments do not seem to hit the lower or upper corners on their apparent prolongation.

Recently, many illusions have been discovered (e.g., Vicario's rarefaction and sloping steps illusions, Gerbino's hexagon masked by triangles, Pinna's angularity illusion, Bressanelli and Massironi's adjoining trapezoids—see Figure 3). So, why do most authors keep brooding over the same invariant small set of core illusions of Figure 1? Is this core a historical accident, and is it still legitimate today?

**Figure 4:**
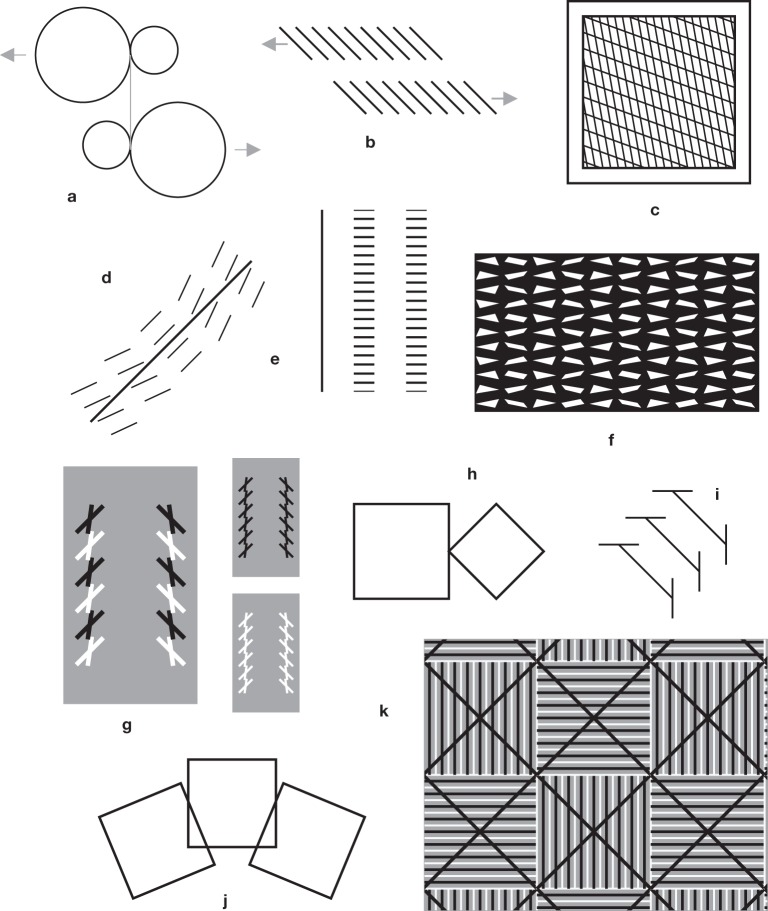
**Figures with illusory effects, found or devised by the author**. **(a)** A figure with four circles having a common vertical tangent. The large circles seem to be pulled toward the left and right borders of the page. (From Ninio, 1979). Constructed as a variation on the illusion with circles of Lipps (1897) (Figure 2h). **(b)** A related effect with Zöllner stacks. The oblique segments are collinear two by two, but the upper ones seem to be pulled to the left with respect to the lower ones. **(c)** A tilt illusion (Ninio and Pinna, 2006). Here the originality lies in the fact that there are two familes of lines at different orientations within the perceptually distorted square. **(d)** The straight line appears to be bent, due to the influence, in the absence of contact, of the oblique segments (from Ninio and Pinna, 2006). In part, the illusion may be due to the principle shown in **Figure 5e**. **(e)** misperceived intervals. The interval between the two vertical stacks appears to be larger than the interval between the vertical line and the left stack (serendipitous observation). **(f)** One sees narrow stripes of alternating black and white elements at a close to 15° orientation. The white elements are real, the black ones are part of the background. The alignments are perfect, yet the ribbons seem to undulate (From Ninio, 2001). **(g)** A hybrid between the twisted cord and the Zöllner illusion. The two stacks on the left with alternating black and white elements seem to converge at their top, contrary to the all-black or all-white Zöllner patterns shown on the right (from Ninio and Pinna, 2006). **(h)** The diagonal of the diamond seems shorter than the side of the square, but they are equal (a variation on a pattern shown in Ninio (1998). **(i)** A hybrid between the Zöllner and the trapezium illusions (from Ninio, 1998). **(j)** The central square has a trapezoidal appearance (a simplified variant of Pinna's illusion of Figure 3k). **(k)** The black lines are at strict +45° or −45° orientations (from Ninio, 2002).

I add here a collection of stimuli that I have produced over the last 35 years (Figure 4). There is, I feel, something to learn from each of these stimuli. With two exceptions (Figures 4e,f) none of them was discovered. They were all designed rationally in attempts to probe the limits of current explanations, find counter-examples, or construct examples predicted by my own theories. However, some readers may judge these stimuli as obvious variants of stimuli previously known to them.

## Measurement theories of illusions

### Mapping distortions

One broad analogy, whether pertinent or not, to understand the origin of geometrical illusions is that of the construction of geographical maps. In order to represent the surface of the earth on a planar map, the geometer must choose a system of projection that inevitably distorts the frontiers. Nevertheless, the construction of the map follows very strict rules. Knowing them, the navigator extracts from the map all the information needed to determine his itinerary with precision. There are many variations on the theme of mapping. In my initial theoretical proposal (Ninio, 1979), the brain has, metaphorically, two instruments, a biased meter that provides a systematic overestimation of large extents, and a more reliable compass for the orientations.

Contrary to what I held for a long time, the visual representation of a figure cannot be entirely assimilated to a map, because it depends upon the way it is looked at. Morinaga's paradox (e.g., Morinaga and Ikeda, 1965; Figure 3i) is a classical counter-example to a strict mapping theory. The study of variants of the Ebbinghaus illusion (shown in Figure 1j) provide another striking counter-example to mapping theories. When the circles are replaced with other elements, strange things happen (e.g., Coren and Enns, 1993; Rose and Bressan, 2002). The homogeneity or non-homogeneity of the surrounding elements, their similarity or non-similarity with respect to the central element play a role. Furthermore, it is even suggested that there are top-down influences in the illusion, and that the semantic similarity between the surrounding and the central elements is also an important factor.

### A basic metric rule: the convexity principle

**Figure 5:**
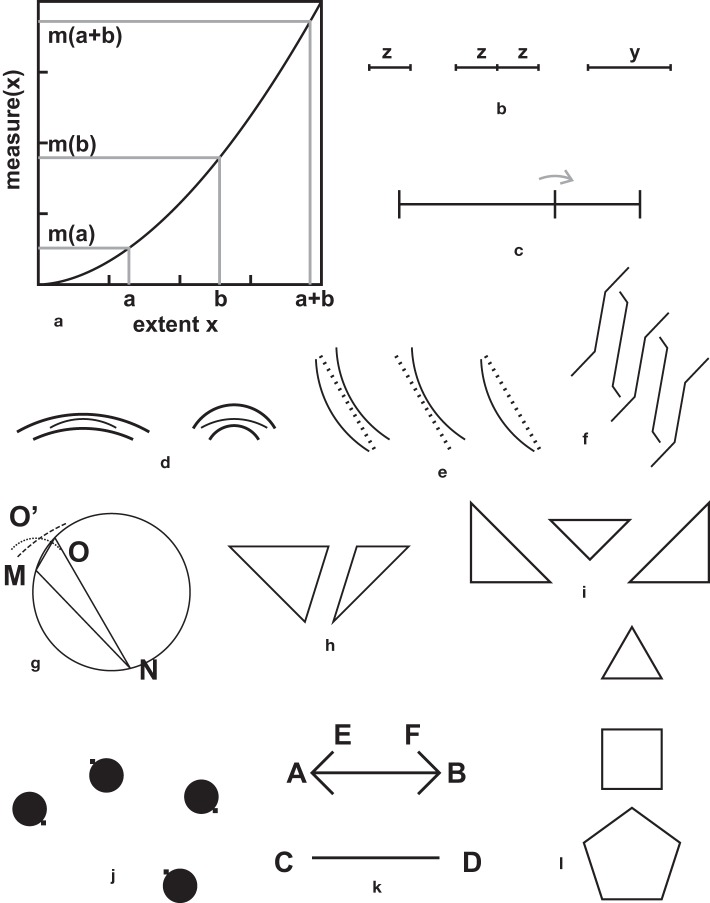
**Convexity illusions**. **(a)** A convex function that goes through the origin. If there is such a relationship between extents in a figure, and their “measured” or perceived values, a number of effects, usually attributed either to contrast, or to assimilation can be predicted. **(b)** Bisection illusion. The undivided segment on the right seems to be larger than the divided segment in the center, but they are equal. “Inverse” bisection illusion: the isolated segment on the left is equal to the half-segments in the center, yet it seems to be shorter. **(c)** According to the convexity principle, a point between two points or two lines should be attracted toward the closest target. The illusions in **(d–f)** seem to follow this pattern **(d)** is an illusion by Lipps (1897). The two central arcs are identical **(e)** is an illusion by Oyama (1960). The dotted lines are straight, but seem to be curved toward or away from the circles **(f)** is an illusion by Lipps (1897). The central parts of the Z-shaped and S-shaped lines are all parallel, but the extremities of the Z-shaped segments seem to be attracted to the closest neighboring lines. **(g)** A tentative explanation of the triangle illusion of Figure 3g. MON is the scalene triangle. When O moves on the circumference, the angle in O remains constant. The perceived shape of the triangle can be estimated by considering that the small side MO is underestimated, and the large side NO is overestimated, with respect to the base MN. Keeping the base MN, the point O would be shifted to the position O' obtained as the intersection of a circle centered on M, with a radius shorter than MO, with a circle centered in N, with a radius larger than NO. They intersect outside the circumference, therefore the perceived angle MO'N is underestimated with respect to the original angle MON. **(h)** Two triangles extracted from Sander's parallelogram (Figure 1e). The illusion is already there. **(i)** A variation, by the author, on the theme of sides and diagonals. The hypotenuse of the central triangle seems to be shorter than the sides of the flanking triangles, but they are strictly equal. **(j)** Naito and Cole's gravity lens illusion. The small squares form a parallelogram that appears distorted due to the pulling or pushing effects of the black disks. The pulls or the pushes are as predicted by the convexity principle: disks with small separation come closer and disks with wider separation are pulled apart. **(k)** See main text, Section A Basic Metric Rule: The Convexity Principle **(l)** The sides of all three polygons are equal, yet they seem to increase from the triangle to the pentagon. The triangle-pentagon illusion was described by Müller-Lyer (1889) and Day (2010) added a square. Squares and pentagons offer the opportunity to have larger diagonals or internal segments than the triangle. Their sizes are therefore overestimated with respect to the size of a triangle, according to the convexity principle. The illusion on the sides of the polygons would be a side-effect.

Assume that you are on the sea front, and you wish to represent the layout of a number of floating targets. Your only instrument is a chronometer. You measure the time it takes to swim from one target to the other. When two targets are close, one can swim rapidly from one to the other. When the targets are distant, one swims less rapidly, and the swimming speed diminishes as the targets become more and more distant. Thus, the measured time to connect two targets grows more than proportionately with the distance between targets. This time, provided by the chronometer, overestimates large distances with respect to small ones. In psychological language, it increases the contrast between large and small. In mathematical language, the measure is a convex one. The relationship between an extent x and its measured value m(x) can be represented by a parabola, or any curve starting at the origin, and rising with a curvature of constant positive sign. (See Figure 5a).

Delboeuf (1865) hit upon a similar formalism. He proposed that the perceived extent between two targets could reflect the duration of eye-movements from one target to the other, including the times for initiating and terminating the movements. He thus proposed a mathematical model in which the measured values would be the sum of three terms that predicted a concave, instead of a convex measure. Delboeuf focused on stimuli other than those I am considering in this Section A Basic Metric Rule: The Convexity Principle. In his article, there is a panel with 34 figures (reproduced, for instance, in Da Pos and Zambianchi, 1996, p. 78, and Vicario, 2011, p. 63). Delboeuf introduced his theme with Figure 14 of the panel, similar to Figure 5c here, but he proposed the opposite effect. He illustrated his principle with Figures 15–19 of his panel, but there are, in my opinion, other principles at work in his examples. Day (2006) conjectured that “it is conceivable that (…) the amount of time it takes to move from one place to another is a determinant of the apparent distance between them.”—but he did not speculate on the law relating space and time.

In an early, contribution (Ninio, 1979) I proposed that a convexity bias was a basic theme in metric illusions. The chronometric metaphor is proposed here to show that a measurement process may provide raw non-linear readings, in particular readings in which the measured value m(x) varies more than proportionately with the measured extent x (Figure 5a). Eye-movement theories might suggest an opposite rule, in which m(x) varies less than proportionately with x. More generally, various theories might suggest various possible relationships between an extent x, and its measured (perceived) size m(x).

Now, in the chronometer and floating targets metaphor, all goes well as long as you consider apparent distances between single pairs of targets. But what if you have at least four floating targets, and wish to put them on a map? You will always be able to draw a quadrangle in which each of the four sides is proportional to its measured value. Then, the lengths of the diagonals on your drawing may be shorter or larger than predicted from their measured values. Some corrections are necessary, and the simplest is to construct the representation by making reasonable compromises when there are discrepancies. So far, there is no bold hypothesis here.

In practice, I found that the combination of a convexity rule for producing a shape and a principle of compromise when there are discrepancies between the measurements accounted well for a large number of geometric illusions in single, non-subdivided figures. They account for some illusions usually explained with an “assimilation” principle (Pressey and Murray, 1976). For these figures (for instance, the Müller-Lyer, or the Sander parallelogram) it would seem that we have just replaced one description by another, equivalent one. However, the underlying reasonings are quite different, and there is at least one illusion (the Delboeuf illusion with circles of Figure 1f) that is well explained by assimilation, but not by convexity.

If we take the simplest figure: a segment joining two points, for which there would be a single measure, it is not clear how we might detect an illusion, if there is one.

In figures with three points, there are several possibilities. First let us consider the “bisection illusion” (Figure 5b). An undivided segment of length y is split into two equal halves of length *z* = *y*/2. The bisected segment looks shorter than the undivided one. But when the small undivided segment of length z is shown close to the divided segment, of length *y* = *z* + *z*, the isolated segment appears shorter than each of the two halves of the bisected segment. How can the bisection of a segment make it appear shorter in relation to the whole segment, and larger in relation to its components? Is this a logical impossibility? I proposed (Ninio, 2011a) that the illusion is not where people see it! There would be a hidden “convexity effect” that makes us represent the large segment *y* more than twice as long as the isolated half-segment, *z* but this is not detected as an illusion. Assume that *z* receives the measure 10, and *y* receives the measure 30. A problem arises with the subdivided segment, because there would be two conflicting measurements. Measured in a single jump from end to end, we get *y* = 30, and measured in two jumps, we get *z* + *z* = 20. So, let us make a compromise such that the measure of the subdivided segment is intermediate between 20 and 30. I this way, the two effects illustrated in Figure 5b are like two faces of a same coin. The figure would be a “pedagogical device” that make the existence of a geometrical anomaly obvious, without telling us the nature of the anomaly.

Still, the story must be more complex, since (i) if a segment is split into two unequal parts, the relationship between one of the sub-segment and an external segment equal to this one is subject to strange variations (Botti, 1906; see also the detailed experimental study of Künnapas, 1955 on the unequal partitioning of the horizontal segment in a “T” configuration).

If a point B is on a segment AC, but not exactly in the middle, it should, according to the convexity principle, be attracted to the closest extremity of the segment, as shown in Figure 5c. An attraction to the closest line is found in several “contrast” illusions, for instance the illusions of Figures 5d–f. A related situation is that of three lines diverging from a same point. Orbison (1939) wrote: “If the center line is moved so that it is no longer midway between the two exterior lines, it will no longer be in a position of equilibrium and will be distorted toward the nearer line.”

Next, if we consider three points A, B, C forming a right angle such that side AB is larger than side BC, and we complete the figure to form a rectangle, we would state that rectangles are perceived as more elongated than they really are, in agreement with Piaget and Denis-Prinzhorn (1953). Then, and this is more subtle, if we take three points and join them to form a triangle, we get the new triangle illusion described by Kennedy et al. (2008), shown in Figure 3g. According to the authors, “For a reference angle of 60°, angles embedded in isoceles triangles were judged to be on average 14° larger than angles embedded in scalene triangles.” Understanding why this is so requires some delicate but unambiguous geometrical reasoning (see the legend to Figure 5g). In my opinion, Sander's parallelogram illusion (Figure 1e) can already be detected at the level of its triangle components (Figure 5h). The same applies to the earlier Dr. Fee's illusion (Figure 2c). This inspired me to construct the pattern of Figure 5i with rectangular triangles. The hypotenuse of the small triangle appears shorter than the sides of the large triangles, although they are all equal.

In figures with four points (quadrangles) we obtain without difficulty the illusion of Figure 3o described by Rausch (1952), in which a parallelogram seems to be larger than a rectangle having equal side and height. We also obtain the Naito and Cole (1994) “gravity lens” illusion (Figure 5j). The small squares form a parallelogram that appears distorted due to the pulling or pushing effects of the black disks. The pulls or the pushes are as predicted by the convexity principle: disks with small separation come closer and disks with wider separation are pulled apart.

The much studied Müller-Lyer appears to me as another instance of the convexity + compromise effect, applied to quadrangles. Assume for instance that AB or CD in Figure 5k is larger than the horizontal distance between the tips of the fins EF (Müller-Lyer pattern with ingoing fins). According to the assimilation principle, the perceived length of AB is “blended” with that of EF, so the apparent length of AB is underestimated with respect to that of the isolated segment of equal length CD. Within the convexity + compromise framework, both AB and CD are overestimated with respect to EF, according to a convex measure. Then there would be a compromise between AB and EF, under the constraint that the orientations of the fins are roughly preserved.

Day (2010), completing an earlier observation of Müller-Lyer (1889), stated that lines of a same length, forming a square appear intermediate in length between those of a triangle and those of a pentagon (Figure 5l). This is again obvious here, as a result of compromises between the measurements of sides and those of diagonals. There would be a hidden effect, the overestimation of the diagonals with respect to the sides, but this would not show up in the closed versions of the square or the pentagon.

**Figure 6:**
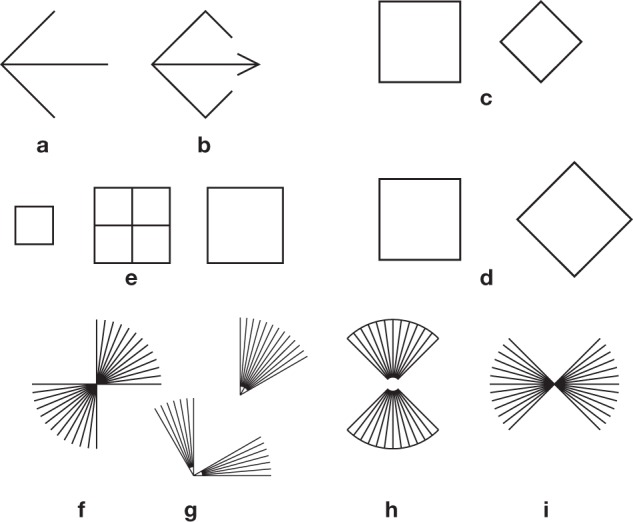
**Some expected and unexpected effects**. **(a)** Pinna's “illusion of the diagonal.” The diagonal seems to stick out of the virtual square. **(b)** A variation on this illusion. The diagonal with an ingoing fin also sticks out of the square, contrary to what one would expect from a Müller-Lyer pattern with ingoing fins (Figure 5k). In **(c)**, as in Figure 4h, the diagonal of the diamond appears to be shorter than the side of the square. This can be taken as evidence against the generality of the square-diamond illusion **(d)**. **(e)** Bisection illusion with squares. Here, the undivided square on the right seems to be smaller than the subdivided square in the center, and the isolated small square on he left seems to be larger than the small squares within the large one in the center. See also the hybrid case of Figure 3n. **(f)** An illusion by von Helmholtz (1866). The bundle of lines seems to cover an angle larger than 90 degrees, suggesting the existence of an expansion effect, as with Helmholtz' squares (Figure 1h). But the examples **(g)** from Tolanski (1964) and **(h)** from de Savigny (1905) seem to suggest the opposite rule. I am undecided about **(i)**.

However, in an open version of the square, allowing the diagonal to stick out, a diagonal does appear to be larger than it should, with respect to the sides. This is Pinna's “illusion of the diagonal” (Pinna, 2003), shown in Figure 6a. In a variant of this illusion, a Müller-Lyer pattern with an ingoing fin, placed on the diagonal of a square, appears subject to expansion, not shrinkage (Figure 6b).

If we compare two unequal squares, in which the side of the large square is equal to the diagonal of the small one, this diagonal is clearly underestimated with respect to the side of the large square (Figures 4h, 6c; Ninio, 1998, 2011b). The opposite would have been expected from the “square-diamond” illusion (Figure 6d), in which there is a similar arrangement of the two figures, but the square and the diamond have equal sizes.

As we see, the convexity principle accounts for a large number of geometric illusions (Figures 5, 6) that are usually explained by a contrast effect (e.g., Figures 5d–f) or, when complemented with a principle of compromise between discordant measures, by an assimilation effect (e.g., Figures 1b,e, 5l). A number of illusory effects cannot be described within this framework, in particular those of Figures 1f–1k, and Figures 1m – 1p. I also mention here, in relation to the bisection illusion (Figure 5b) that if segments are replaced with squares as in Figure 6e, the apparent sizes of the squares vary in the opposite direction: the central subdivided square seems larger than the undivided square on its right, and its components appear smaller than an equal isolated small square shown on the left (Ninio, 2011a)! However, there is also the possibility that rules for single lines may differ from rules on objects, a divided square counting as an object.

### Stretching rule for subdivided figures

Returning to the chronometer and floating targets metaphor, what happens if a small number of targets form a contour of which we wish to understand the shape, and there are many intermediate targets between pairs of contour targets? In the simplest case, we have two well separated targets and a string of intermediate targets between the two. According to the convexity rule, the apparent distances between two consecutive targets is underestimated with respect to the distance between the terminal targets. Therefore, if the distance between the terminal targets is estimated by adding the measures of the distances between nearest-neighbor targets, it may be severely underestimated. Thus, it is reasonable to expect that, in a measurement framework, there would be a corrective factor that produces an apparent expansion of subdivided lines. In practice, an expansion of subdivided segments is observed when the number of intermediate points is *n* ≥ 4. This is shown in the “Oppel-Kundt” illusion (Oppel, 1855), of Figure 1i. There is also the perhaps related Helmholtz lined square illusion (von Helmholtz, 1866) in which a virtual square formed of several segments parallel to one side is perceived as stretched in the direction orthogonal to these segments (Figure 1h), and the related fan illusion (Figure 6f) in which a bundle of straight lines constrained within a vertical and a horizontal axis seems to expand beyond the 90° angle.

**Figure 7:**
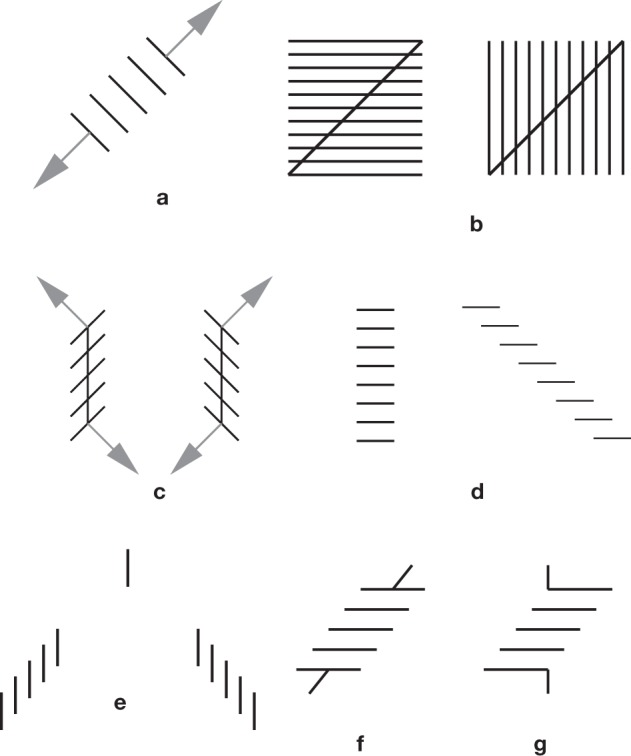
**The orthogonal expansion principle**. **(a)** A classical effect, the expansion of subdivided figures, as observed in the Oppel-Kundt illusion, and the Helmholtz square illusion. **(b)** The effect of this expansion is shown on Helmholtz squares. There is a side-effect on the orientations of the diagonals. This effect nicely agrees with a perceptual enlargement of acute angles, always present in the illusions of the Ehrenstein-Orbison family. For a more elaborate variant of **(b)**, see Figure 4k. **(c)** orthogonal expansion effect. It is suggested that with a stack of segments piled up obliquely, the expansion occurs orthogonally to the segments. This is the orthogonal expansion principle of Ninio and Pinna (2006). **(d)** The oblique side on the right, as noticed by Botti (1909), appears more expanded than the central stack, suggesting that orthogonal expansion might work better with oblique than with straight stacks **(e)** is a variant of the Zöllner illusion studied by Ninio and O'Regan (1996). The isolated single segment in the center belongs to both stacks, yet it seems to be much higher than the meeting point of the stacks. The distortion of the stacks cannot be described by a rotation, because all the segments have their vertical orientation preserved. The distortion is however fully compatible with the orthogonal expansion principle. **(f)** A Poggendorff-Zöllner hybrid, shown in Ninio and Pinna (2006). The two segments at the end of the Zöllner stack are collinear. There is a perceived misalignment effect, and it is as predicted by a Poggendorff illusion. **(g)** Here, two segments are abutting orthogonally on the terminal segments of a Zöllner stack. The two abutting segments are collinear. I find no illusion.

There is undoubtedly a stretching effect in subdivided figures, but it may be rather subtle. The Helmholtz square illusion may be an instance of a more general effect of “orthogonal expansion.” The idea is that when there are several parallel or nearly parallel segments, there is an expansion in a direction that is orthogonal to the segments. When the segments are piled up obliquely, as in a Zöllner stack, this orthogonal expansion generates an apparent rotation effect. This is illustrated and explained in Figure 7 and its legend. It was suggested by experimental observations on the Zöllner illusion, and related patterns.

### Additional rules

**Figure 8:**
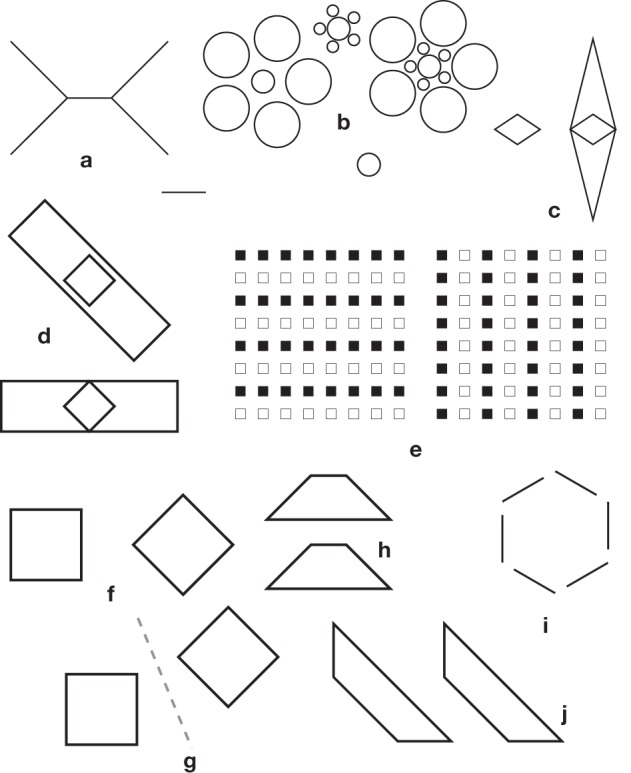
**Miscellaneous effects**. **(a)** Reversal of the Müller-Lyer illusion when the outgoing fins become very large (compare the shaft with the isolated segment on its bottom right). **(b)** Variations on the Ebbinghaus illusion. **(c)** The small rhombus on the left appears to be slightly larger than the enclosed rhombus on the right. **(d)** The diamond at the bottom, within a horizontal rectangle, is perceived as a square when it is enclosed by a rectangle oriented at −45° (Kopferman, 1930). **(e)** The left figure with horizontal alignments of black squares appears to be compressed vertically, contrary to the expectations from both the vertical-horizontal illusion and Helmholtz square illusion (Albertazzi and Pinna, 2008). **(f–i)**. Asymmetries in illusory patterns. In **(f)** the diamond “points” at the square, enhancing significantly an effect that is already observed when the two figures are placed symmetrically **(g)**. In **(h)** one trapezium “points at the other,” as in Shepard's table illusion (Figure 3a), and the Bressamelli and Massironi illusion (Figure 3j). In **(i)**, a variant of Gerbino's illusion (Figure 3f) without the masking triangles. All the segments belong to a hexagon. In **(j)** we can see the trapeziums as twisted ribbons, the vertical segments being at the back, and the horizontal ones protruding toward us (e.g., Ninio, 2011a).

The convexity + compromise rule, even when complemented with an orthogonal expansion rule fails to account for a number of metric illusions. Among these there is the Ebbinghaus illusion (Figures 1j, 8b) and the reversal of the Müller-Lyer illusion when the outgoing fins become very large (Figure 8a). Since the convexity rule tends to overestimate large extents, it would be reasonable to apply a corrective factor that works in the opposite direction—a factor that would reduce the perceived overall size of large figures (Ninio, 1979). For instance, there would be in the case of the Ebbinghaus illusion an enlargement of the whole group with small circles, and a shrinkage of the whole group with large circles, but this would not be detected as an illusion. However, up until now, I failed to construct an example showing this hypothetical effect. Furthermore, in the variant of Figure 8b in which a central circle is surrounded by a close band of small circles, and an outward band of large circles, the central circle appears somewhat larger than in the variant with large surrounding circles exclusively. Note also that the Ebbinghaus illusion is known to be modulated by attention, as observed in people from remote cultures (e.g., de Fockert et al., 2007). Furthermore, there are many studies showing that if the surrounding circles are replaced with different shapes, the illusion may be strongly altered (see Section Mapping Distortions).

Therefore, it would seem that if there is a space occupation rule, it is of very limited applicability. In fact, there is evidence (pointed to me by Giovanni Vicario) in favor of a rule saying that a figure enclosed by another figure appears slightly smaller than when it is isolated. For instance, in Figure 8c, the isolated small rhombus on the left appears slightly larger than the enclosed rhombus on the right. This phenomenon might be related to the apparent shrinkage, of occluded figures, studied by Vezzani (1999). On the other hand, it has also been held (at least, in the case of segments enclosed by a rectangle) that enclosed patterns look larger than their isolated counterparts (Fellows, 1968).

Another source of complication arises from the fact that some figures may induce privileged ways of being inspected, that may differ from the dominant vertical and horizontal axes. For instance, a diamond enclosed by a rectangle may be perceived either as a square, or as a diamond, depending on the orientation of the enclosing rectangle (Figure 8d), as shown by Kopferman (1930). Albertazzi and Pinna (2008) constructed a number of patterns for which there may be privileged inspection directions, generating unexpected illusions (Figure 8e).

I am also struck by the fact that many illusions involve two identical figures that are oriented differently. One figure, so to speak, “looks at the other.” This is clear in the case of Shepard's table (Figure 3A) and the square-diamond illusion (Figures 8f,g). There is also an asymmetry in the Bressannelli and Massironi illusion (Figure 3j) and in the trapezium illusion—here the upper trapezium “embraces” the other (Figure 8h). In Figure 8i I show a variant of Gerbino's hexagon illusion of Figure 3f, but without the usual occluding triangles. The bare segments are arranged asymmetrically, for instance the top left segment is pointing toward one terminus of the top right segment, and the set of six segments does not seem to be on a hexagon (Figure 8j) is an anecdotical but very striking effect with trapeziums: Here, we can see the trapeziums as twisted ribbons, the vertical segments being at the back, and the horizontal ones protruding toward us (e.g., Ninio, 2011a). I have often projected this figure on large screens, at public conferences. People may take time to see the 3d effect, but when they get it, they may have difficulties to revert to the 2d interpretation.

### About anisotropy effects

Most specialists will agree with the proposal that the vertical-horizontal illusion is a primary visual effect. It is a factor that contributes to many visual patterns, so the illusions they produce must be corrected by the vertical-horizontal effect. It is strongly subject-dependent. I might have stated that our view of a scene is subject to an anamorphosis that expands the scene in the vertical direction, but this is probably not true. For instance, although a square looks elongated along the vertical direction, a circle does not. It does not look like an ellipse elongated along a vertical diameter—or does it? This point deserves to be settled experimentally. I believe that a circle does look like a circle, that we have perhaps a kind of instrument to measure curvature that would tell us that curvature is the same all along the circumference of a circle. Here, I am introducing an important theme—that the illusory effects of which we are aware are those that remain after being somewhat counteracted by control instruments.

There is a striking anisotropy effect, the square-diamond illusion. A classical interpretation of this effect is that we appreciate the dimension of a square according to the lengths of its sides, and that of a diamond according to the length of its vertical and horizontal diagonals. However, as I showed experimentally (see Section Trapezium, Square-Diamond and Other Illusions) (i) the illusion is due, in part, to an asymmetric presentation of the square and the diamond: the diamond “points” at the square, but the square does not point at the diamond. When the square and the diamond are presented symmetrically, the illusion is substantially reduced (Figures 8f,g) (ii) the illusion can be dominated by a stronger effect, in which a side of a square looks larger than the diagonal of a diamond of the same length (Ninio, 2011b, see again Figure 6c). I add here that there is a general cultural problem with patterns composed of two very similar figures. People may apprehend the patterns globally, or they may fixate their attention on one figure then the other. They may also have culturally privileged viewing axes that favor symmetry perception and that differ from the horizontal and the vertical axes.

Are there anisotropies in the perception of orientations? It is often considered that there may be two tendencies: (i) a tendency for orientations to be perceived as closer to the vertical than they really are (Hotopf, 1981). This would agree with a vertical magnification effect, but I am not aware of a quantitative treatment reducing one effect to the other. It would require precise data, due to the fact that the horizontal-vertical illusion is strongly subject-dependent. (ii) there would also be a tendency for orientations close to the horizontal, to be perceived as closer to the horizontal than they are. Rochlin (1955), quoted by Stuart and Day (1988) has shown “that parallel lines do not appear parallel at all orientations (…),”and that “parallel lines are distorted away from parallel when tilted clockwise from vertical, but not when they are tilted counterclockwise from vertical.”

### On paradoxes in shape perception

What we find strange, and sometimes classify as an illusion, turns out to be a legitimate perception once we understand better the geometry of space and shape. One historical example is the observation of Lucretius about the appearance of a monument as seen in perspective, which he reported as being an illusion, while we accept this as a legitimate perspective effect that does not require an explanation. (Quite to the contrary, we feel that we need to explain the anti-perspective effect of size constancy at short distances).

**Figure 9:**
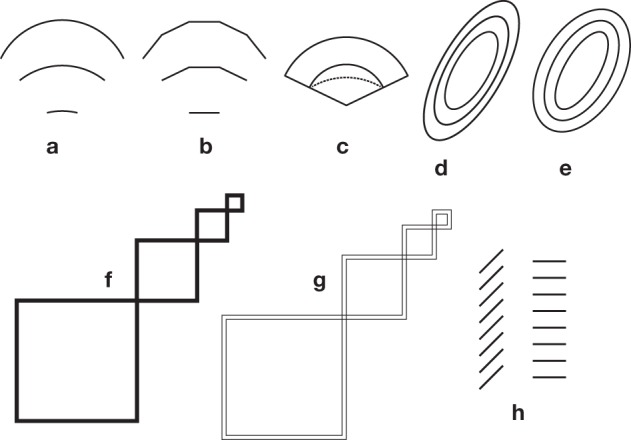
**Paradoxes of shape perception (see the Section On Paradoxes in Shape Perception)**. In **(a)**, from Ninio (1979). Just below an arc of 120° is shown a fragment of this arc covering an angular sector of 60°. Still below, one sees a still shorter arc. The shorter the arc, the flatter it looks, yet all three arcs have the same radius. In **(b)** the arcs are approximated with broken lines, and one cannot speak of an illusion **(c)** The two continuous black arcs are concentric. They have the same center and different radii. One arc is thus a reduced variant of the other, they are seen equally curved. The arc represented with a dotted line is a truncated version of the arc above (they have the same radius) and it is seen as flatter as the arc just above, which is geometrically legitimate. This arrangement shows that the dotted arc must indeed be seen as flatter than the top continuous arc. **(d,e)**, from Ninio (2011a). The ellipses in **(d)** are homothetical, those in **(e)** are equally spaced. The curves in **(e)** appear more similar to each other than the ellipses in **(d)**. **(f,g)**, A variation on an illusion by Ehrenstein (1954), shown in Ninio (2011a). In **(f)**, when one goes from the larger square to the smaller ones, their outlines seem to become thicker and thicker, yet they are rigorously of the same width. Since the line thickness is constant in **(f)**, the ratio of the area covered by the lines to the blank area of the squares does increase as the size of the squares decreases. Small squares have indeed (relatively) thicker borders. A similar effect can be detected with the small circles in Figure 1j. In **(g)** the (bold) contour of the squares is replaced with a pair of thin, parallel outlines. The space between the parallel contours seems wider when the squares are small. In **(h)** the segments are equally spaced along the vertical in the two stacks, yet there seems to be a strong compression effect in the stack with the oblique segments. This is probably not an illusion, if we measure the distances between the segments orthogonally to the segments.

As a general rule in shape perception, we accept the principle that the perceived shape of a figure does not depend upon its size: A figure can be enlarged or reduced, and look the same all the time. This creates some problems in the perception of curvature because curvature—contrary to angles—varies with size. This is illustrated with the so-called flattening of short arcs effect, already discussed in Ninio (1979) (Figure 9a). In this stimulus, three arcs having the same radius but different extensions are shown one above the other. The widest arc is on top, the narrowest one is at the bottom. The shorter the arc, the flatter it is perceived. Actually, the three arcs are not a same object at different sizes, but an object and its truncated versions (as in Figure 9b). When we say that an arc is more or less flat, we refer to a shape property. However, shape should not be invariant under truncation (Figures 9b,c), so the three arcs in Figure 9a need not be perceived as equally curved. One reviewer remains unconviced. He writes: “At the most basic framing of the illusion, one mentally moves one arc on top of another and decides if they match, or if the shorter arc diverges somehow. For me, there is a clear effect even at that level. Is that not an illusion?”

I do not wish to be dogmatic about shape invariance under homothetic transformations. The ellipses in Figure 9d are homothetic, those in Figure 9e are equally spaced, and decidedly not homothetic. The curves in the second set (there is a single true ellipse among them) appear more similar to each other than the ellipses of the first set.

Another intriguing effect, described by Ehrenstein (1954) can be counted as a similar paradox of shape perception. Empty squares differing in size are drawn with contours of equal thickness. The smaller the figure, the thicker its contour appears to be. This is again legitimate from the point of view of shape perception. The surface occupied by the contour in the small square is relatively larger, compared to the inside surface, than it is in the larger square. The paradox is enhanced in the stimulus of Figure 9f, by connecting the small and the large squares by contour lines of constant thickness. In this figure, there are two conflicting principles at work. The veridical perception of line thickness implies a perception of constant line thickness. The respect of shape constancy makes us perceive the thickness of the contours in relation to the size of the enclosed figures. A similar argument applies to the stimulus of Figure 9g. Here, the thick contour lines are replaced with thin double contours. The separation between the double contour lines is invariable, yet it seems to be larger when the double contours enclose a small square.

In Figure 9h, there is on the left a stack of oblique segments, piled up vertically, and on its right a stack containing horizontal segments of the same length, also piled up vertically. The vertical separation of the segments is exactly the same in the two stacks, but there seems to be a strong compression effect in the stack with the oblique segments (e.g., Judd, 1899). Morgan and Casco (1990), argued that this might be a pure “trigonometric” effect. What counts, for the perception of spacing between adjoining segments, in this case, would be their distance, taken orthogonally to the segments, a choice agreeing with geometer's practice. There would be a perceptual compression effect in the left stack if we appreciate the spacing along the axes of the stacks, i.e., here, in the vertical direction.

## The pertinent classification issue

Classification has been of paramount importance in several domains of science. Linnaeus classification work on plants paved the way for both Lamarckian and Darwinian theories of evolution (although Linnaeus himself was a fixist). Mendeleev's classification of the natural elements, embodied in his famous table, paved the way for the understanding of the electronic structures of atoms, and from there, to large sections of modern chemistry and physics. A major difficulty, in a classification task, is to neglect some features that jump to the eyes, and focus on less obvious aspects that turn out to be far more pertinent. In the classification of natural elements, the physical state—solid, liquid or gaseous—turns out to be of secondary importance, while the types of chemical bonds that the element can make is of the highest importance. In the classification of species, living on land or in the sea is secondary, while the sexual reproduction mode is primordial (thus, a dolphin is not a fish).

In a classification of geometric illusions based uniquely upon their appearance, one may distinguish two major groups: metric illusions, and orientation illusions. The metric illusions are often split into two categories: contrast illusions, and assimilation illusions. Metaphorically, the brain would use two instruments: a meter and a compass. But how to classify an illusion when an angle looks wrong? Should we invoke a metaphorical third instrument that measures angles,—a protractor —used by the brain? The protractor would produce biased measure of angles—the so-called “regression to right angles” tendency (Wundt, 1898). Day (2010) treats Poggendorff, Zöllner and Müller-Lyer as instances of a same basic effect, whereas most other authors treat them as distinct effects.

I believe that it is very important—as a substitute for brilliant insights—to develop objective classification criteria, based upon experimental paradigms. There were great hopes that the stereoscopic perception of illusory patterns could produce clear-cut results. Seymour Papert (1961) constructed a random-dot stereogram representing, in camouflaged form, a Müller-Lyer pattern. This pattern could not be perceived monocularly, but clearly emerged in depth under stereoscopic viewing. Since the pattern was not accessible to the brain prior to the combination of the optical information from the two retinas, Papert concluded that the illusion arose at a late stage of visual perception, after the stage of combination of the visual streams from the two eyes. Julesz (1971) showed that other geometrical illusions that were camouflaged in random-dot stereograms, were similarly produced under stereoscopic viewing (including Ponzo, horizontal-vertical, Ebbinghaus, and Poggendorff). There was however an exception: the Zöllner illusion (Figure 1m) was not maintained in the random-dot stereogram presentation. Therefore, it seemed, it arises rather early in visual processing, before the stage at which the streams from the two eyes are normally combined.

Thus, it seemed, stereoscopic vision provided an objective way to classify the illusions into two groups, the “early” and the “late” illusions. However, there was a hidden assumption in this work, that was also present in almost all the stereoscopic work of Bela Julesz: that the nature of the pattern is not altered by its encoding in random-dot mode. His stereograms, by construction, did not contain visible edges, contrary to natural scenes in which there are plenty of edges at various orientations. So an effect that required lines with well-defined orientations may not well be captured in a random-dot stereogram. As a matter of fact, Herbomel and I were able to construct stereograms containing camouflaged Zöllner patterns with explicit edges. In this case, the Zöllner illusion was well-preserved in stereo (Ninio and Herbomel, 1990). With a few exceptions, studies on stereoscopic versions of 2d illusory patterns were rare (but see, e.g., Schiller and Weiner, 1972; Wang and Idesawa, 2004). One reviewer objected that RDS studies may be misleading, beacuse “it could as well be that the same illusion can occur early or late depending on how it is presented,” and he/she suggested to “test the temporal resolution of an illusion viewed monocularly vs. binocularly.” The suggestion to study the temporal aspects is quite pertinent. Actually, Rychkova and Ninio (2011) have studied stereopsis under alternating presentation conditions. Incidentally, they reported the existence of ‘intermediate’ 3d percepts at sub-optimal alternation frequencies. The work deserves being extended to stereograms representing illusory patterns.

Another path to an objective classification of geometrical illusions might be provided by the study of their dependence on eye movements. It has long been held that geometrical illusions do not depend on eye-movements, since they are also observed when the stimulus is presented in a flash, too briefly to allow eye-movements. However, Fischer et al. (2003) reported that “some well-known geometrical illusions disappear when the eyes are fixating and saccades are suppressed for a period of time. (…). Any saccade made on purpose restores the illusion immediately.” Some illusory effects disappeared quickly upon fixation, others seemed to persist but finally disappeared. A few illusory effects were stable. Therefore, there is here a promising path to an objective classification of geometric illusions.

There is still another possible experimental strategy that applies to patterns that can be decomposed into two sub-patterns (for instance, the Müller-Lyer illusions with ingoing and ougoing fins). The method is to present the two sub-patterns in rapid alternation on a computer monitor. There is a spectacular effect of shrinkage or expansion on the screen. However, there is no systematic study of such effects, to my knowledge.

Starting in the end of the 1990's, I initiated with Kevin O'Regan a program with the ambition to acquire data that could be useful in an objective classification of geometrical illusions. We measured the magnitude of illusions in various figures at equally spaced orientations, over a range of 360°. We thereby produced what might be termed “orientation profiles.” Comparing the orientation profiles for many illusions and their variants gave us a sensitive technique for judging whether the variants might be related, and whether or not they are likely to be derived from the same underlying mechanisms (Ninio and O'Regan, 1996, 1999). Precise orientation profiles also allow us to decompose an illusory effect into more elementary components, see Section Orientation Profiles.

## Orientation profiles

### General

By “orientation profile”, Kevin O'Regan and I (1996) meant the plot showing how the magnitude of an illusory effect in a pattern varied as a function of the orientation of the pattern. We hoped to obtain, for each illusion a kind of characteristic signature. By comparing orientation profiles of various illusions, we would be able, we thought, to improve the illusion's classification. It is important, in my opinion, to study geometrical illusions at all orientations. Theories about illusory patterns as somewhat related to perspective effects in a “carpentered world” (e.g., Gregory, 1963; Spehar and Gillam, 2002; and many others) become less convincing when one looks at detailed orientation profiles. The discussed illusory effects occur at nearly all orientations. Their magnitude varies smoothly with their orientation. It is sometimes rewarding to look at any illusion just upside down. Our monkey ancestors were living part of the time swinging with their tails wrapped on a branch, and the body upside down—this would explain why the perception of some illusory patterns is not as strongly dependent upon the vertical and the horizontal as required by perspectivist theories.

### Horizontal-vertical illusions

Some orientation profiles had already been published by several authors, in particular for the horizontal-vertical illusion and some of its variants (Hoffmann and Bielchowsky, 1909; Künnapas, 1955; Cormack and Cormack, 1974; Bulatov and Bertulis, 1999). If we rotate a horizontal and a vertical segment in the plane, they exchange their status every 90°. In the intermediate range of rotations (for instance when the original test figure is rotated by 30°, so that one segment is at 30°, and the other is at 120°), there is still an illusory effect. There are also hints that the orientation profiles are less symmetrical with respect to the vertical than expected. Quantitatively, the illusion varies smoothly all around the trigonometric circle. The observed smooth variation may be unexpected for some of us, but it may be rationalized by invoking a general anamorphosis in the vertical direction, so whatever the orientation of a segment, its vertical component would be enlarged. However, I am not aware of the existence of a mathematical treatment of the data that would support or refute this interpretation.

### Müller-lyer

My experiments on orientation profiles with Müller-Lyer patterns were frustrating. I studied stimuli containing Müller-Lyer patterns and visually related stimuli, including the receding arrow illusion (Figure 3h), and Judd's bisected arrow illusion (Figure 1c). Unfortunately, my orientation profile experiments failed to show a relationship between the Mûller-Lyer, the Judd and the receding arrow illusions. The results with Müller-Lyer patterns were erratic. They were strongly subject-dependent, there was no simplifying symmetry when the patterns were turned upside down, etc. My provisional, not too satisfactory, explanation is that a subject may compare the lengths of the segments between the fins according to various criteria, (for instance, forming a virtual rectangle with a pair of segments, looking at orientations, etc.) and the criterion he/she chooses depends upon the orientation of the stimulus. These erratic results are in strong contrast with almost all my other results on orientation profiles. It was perhaps a mistake to study pairs of patterns placed one above the other. I now recommend to study versions of the illusion in which the shafts are placed on a same line. Bertulis and Bulatov (2001) published detailed data on the Müller-Lyer illusion at two orientations; they also performed detailed studies of the Oppel-Kundt and the bisection illusions, and showed their orientation profiles.

### Poggendorff

**Figure 10:**
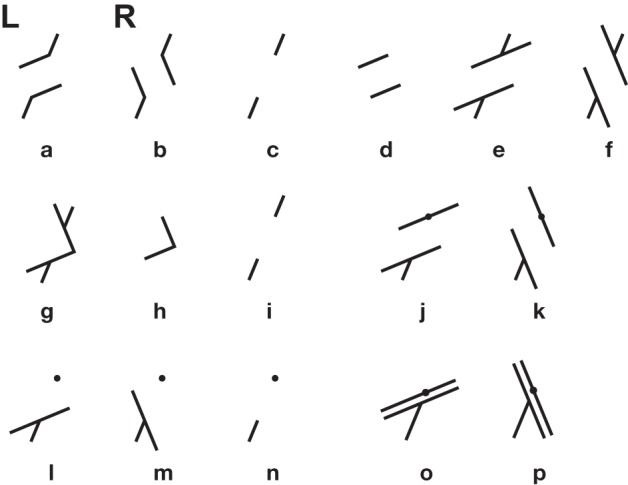
**Poggendorff variants and controls**. Orientation profiles were established by Ninio and O'Regan (1999) for the 15 patterns of this figure **(c,d,h,i,l,n)** are controls for the various corresponding illusory patterns. They were subtracted from the results on the illusory patterns. Data were obtained for the corner-Poggendorff illusion **(g)**, our standard Poggendorff illusion **(e,f)**, the obtuse angle variants **(a,b)**, the illusions with a single oblique **(j–m)**. The patterns in **(o,p)** were studied by Weintraub et al., and some of their results were reproduced in Ninio and O'Regan (1999). The “L” and “R” annotations in **(a,b)** indicate our conventions for distinguishing a pattern from its mirrror-image pattern.

The Poggendorff illusion and its many variants (Figure 10) have generated a considerable amount of speculations, and a reasonable amount of experimental studies. In its standard variant, there are two collinear segments abutting obliquely on two, usually long, parallel lines. The illusion consists in the fact that the two collinear segments appear to be parallel but not collinear, and the direction of their lateral separation is what would have occurred had the segments rotated around the abutting intersections according to a law of enlargement of acute angles. Theories of the Poggendorff illusion usually invoke a rationale based on perspective effects in a carpentered world (e.g., Gillam, 1971; Phillips, 2006), or in natural surrounds (Howe et al., 2005). In another spirit, theoretical discussions have focused on whether the Poggendorff illusion should be due to a metric effect, or to an orientational effects. Greene (1994), p. 666, lists 6 possible components to the Poggendorff illusion that were put forward in 15 publications. The “corner-Poggendorff” pattern (Figure 10g) is particularly interesting because here, the collinear obliques do not seem to be parallel, they do seem to diverge according to a law of perceptual enlargement of acute angles. Vicario and Zambianchi (1993) argue that this pattern is not a Poggendorff variant, but a variant of a pattern proposed earlier by Judd (1899), in which two collinear segments abut on an ellipsoidal shape, and seem to diverge (Figure 2i). I fully agree with them on the equivalence between the Judd pattern and the corner-Poggendorff pattern, but I consider both as variants of the Poggendorff effect.

Quite important and detailed observations were published by Weintraub and co-workers on variants of the Poggendorff illusion (Weintraub et al., 1980). Weintraub and his colleagues studied the simplified pattern composed of two parallel lines, an oblique abutting on one line, and a small disk on the other line, collinear with the oblique (Figures 10o,p) They found that the main determinant of the illusion was the orientation of the oblique—not that of the parallels. They reported orientation profiles for several angles between the oblique and the parallels (from −15° to −90° and from 15° to 90°). They showed that the illusion increased when the angles decreased in absolute values and that there were maxima around the −45° and +45° orientations of the obliques, but not exactly.

**Figure 11:**
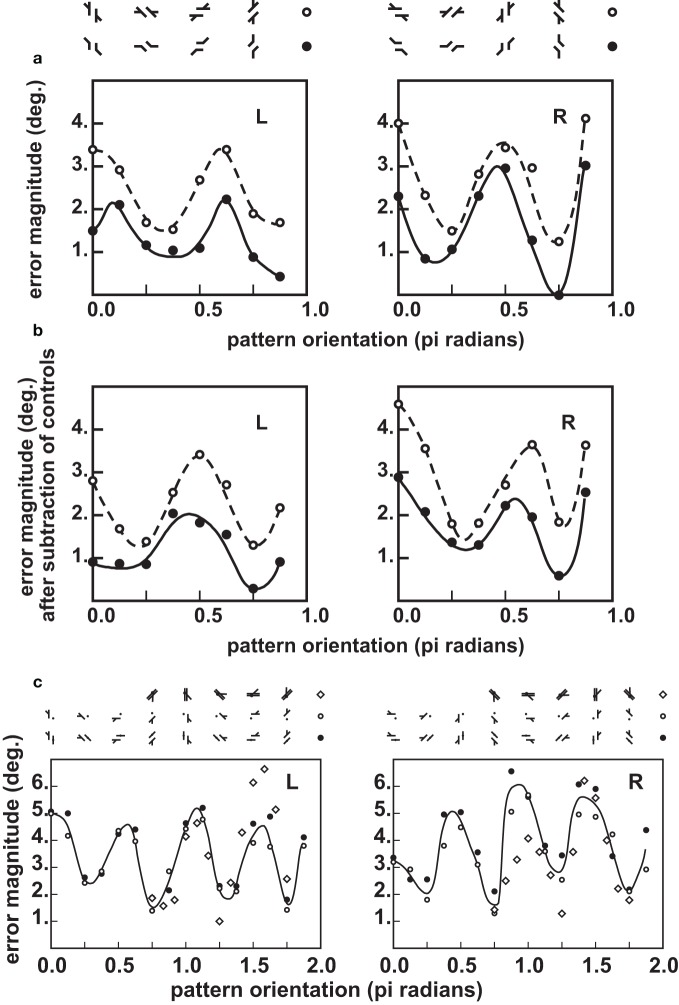
**Orientation profiles in the Poggendorff illusion, and some of its variants (from Ninio and O'Regan, 1999)**. Top panels **(a)** Misalignment measured in the obtuse-angle (Figures 10a,b) and the standard Poggendorff (Figures 10e,f) configurations. The errors are given in degrees, according to a mathematical procedure described in Ninio and O'Regan (1999), applicable in a homogeneous way to all the studied patterns. The results for the L and the R configurations are shown separately. Each data point represents an average over 100 experimental determinations (10 measurements by each of 10 subjects). **(b)** The same results after subtraction of the pure misalignment effect (Figure 10c) are shown in the central panel. Bottom panels **(c)** Misangulation in configurations with a single arm. The results for the L and the R configurations are shown separately, as indicated in the figures. Here, black disks or unfilled circles represent averages of the results for patterns of Figures 10j,m. Each black disk or unfilled circle represent averages over 10 measurements by each of six subjects. The diamonds are replotted from the work of Weintraub et al. (1980), and correspond to one measurement made by each of 48 subjects.

Kevin O'Regan and I reexamined the issue. We determined the orientation profiles for Poggendorff patterns having a 45° angle between the oblique and the parallels, and there were several controls to distinguish between metric and orientational effects (see Figure 10). We confirmed the observations of Weintraub and co-workers and found, to our surprise that the variant they had studied, with a single oblique gave a far stronger illusion than any of the other classical variants (see Figure 11).

We firmly concluded from our orientational profiles that the Poggendorff illusion is mainly a misangulation effect—contrary to my initial hypothesis, developed in Ninio (1979). This effect is observed in its purest form, in the corner-Poggendorff illusion. In this pattern, the misangulation component is always compatible with a trend toward orthogonality. It has a characteristic orientation profile: the maxima are located strictly at the ±45° orientation of the obliques, and the peaks are quite symmetric (Ninio and O'Regan, 1999). In most other variants, the illusory pattern is asymmetric, one can speak of an “L” variant, and its symmetric “R” variant (see Figures 10a,b). In the orientation profile of the “R” variant, there is a maximum near the 45° orientation, whereas the maximum is around 67° for the “L” variant (Figure 11a). The results are contaminated by the “pure misalignment effect” (the Zehender, 1899 illusion, Figure 10i) that tends to increase the illusion at some angles for one configuration, and decrease it by the same amount in the symmetric configuration. After subtraction of the pure misalignment effect, we obtain the profiles in Figure 11b. The maxima are shifted significantly. The magnitude of the illusion is much larger than in the case of the corner-Poggendorff, and it is lower in the patterns with two obliques, than in the patterns with a single one.

Kevin O'Regan and I interpreted the hierarchy of illusion magnitudes as follows. We postulated that parallelism, more than collinearity, is a strong primitive in shape perception. If misangulation plays a role, in the standard Poggendorff illusion, it would affect collinearity judgments but not parallelism judgments in most patterns, because the misangulation effects would be compatible with the maintenance of the parallelism of the two obliques. However, in the case of the corner-Poggendorff configuration, misangulations on the two sides of the corner destroy the parallelism of the obliques. Then, the misangulation effects is counteracted by the influence of parallelism detectors. Now, why does the configuration with a single oblique give a stronger illusion than those with two obliques? Perhaps there is a collinearity detecting device (Field et al., 1993) that works well with segments, thus counteracting the variants with 2 collinear obliques.

### Zöllner when an orientation illusion becomes a metric illusion

I made an erroneous choice, in my theoretical 1979 article (Ninio, 1979). I left aside the Zöllner illusion (being in part, induced in error by Bela Julesz). It seemed obvious to me that the Zöllner patterns had a chiral character (one of the two stacks can be labeled left the other can be labeled right—see Figure 1m). The illusion can be described by saying that one pattern is perceptually rotated clockwise, and the other one is rotated in the anti-clockwise direction. So, I thought that the brain was sensitive to the chiral character of the stacks and that it applied independent transformations to each stack (Ninio, 1977). This was, characteristically a youth mistake, I was very pleased to find, in a phenomenon of shape perception, the imprint of a subtle mathematical notion. However, chiral patterns generating a torque are now invoked—with reason, I believe—in relation to illusions of the Münsterberg family (Figure 2k, see e.g., Wade, 1982; Kitaoka, 1998; Kitaoka et al., 2001).

The Zöllner illusion was studied in conjunction with several variants in a large number of studies. It was often compared with Orbison-like patterns (a target line over a background of parallel inducing lines at a different orientation) or with tilt illusions (a target line surrounded by a band of parallel inducing lines at a different orientation). In particular many such studies were performed in Japan (review in Oyama, 1960), and orientation profiles were published, but they lacked precision concerning the position of the peaks and their shapes. Kevin O'Regan and I were mostly interested by the following question: Is the Zöllner illusion the result of an interaction between stacks of opposite handedness (as it is nearly always represented, for instance in Figure 1m), or can it be already demonstrated in stacks of a single, left or right handedness? The orientation profiles were established for 10 variants, plus some variants of variants. Ironically, the standard presentation of the Zöllner illusion as closely packed, vertically oriented stacks is about the least efficient one, first because the illusory effect is minimal at the vertical orientation of the stacks, then because the illusion increases with the separation of the stacks.

The results were consistent with the existence of a single common mechanism underlying the standard pattern of Figure 1m, and the other patterns, including patterns with stacks of a single polarity, or patterns with two stacks of different polarities, but arranged in a non-classical way (e.g., Figure 7e). Concerning this figure, a reviewer objected “that to see the top middle segment between the two converging lower stacks as part of those stacks would mean that the observer groups that segment with the stacks. I hardly believe this to be the case (…) In addition, consider that eventually, if forced to see the connection between the central segment and either or both stacks, you would have a variation of the Oppel-Kundt illusion, not of the Zöllner illusion.” However, Figure 7e is one of several variants studied in the 1996 article, and the results were highly consistent over all variants and orientations. The points raised by the Reviewer may contribute to the illusion, but cannot be, in my opinion, its major determinants. O'Regan and I suggested that the illusion itself is not a rotation of the stacks but “a shear deformation in which the segments of a stack slide with respect to one another, or an expansion of the stacks orthogonally to the segments.”

In order to have a more precise idea about the nature of the deformation in the Zöllner illusion, consider the pattern of Figure 7f with a single Zöllner stack, and two abutting collinear small segments at the two ends of the stack (Ninio and Pinna, 2006). It can be considered as a hybrid Zöllner/Poggendorff pattern. There is a perceived misalignment effect, and it is as predicted by a Poggendorff illusion. We anticipate that there is also a Zöllner illusion in the stack. But if this illusion amounts to a rotation of the whole stack, we should expect no effect on the positions of the abutting segments. If the illusion amounts to a shear deformation, there should be a disruption of collinearity of the abutting segments, but in a direction opposite to that of the Poggendorff effect. In this case, Zöllner and Poggendorff effects would counteract each other. Last, if there is an orthogonal expansion of the stacks, the Zöllner and the Poggendorff effects would reinforce each other. Now, consider the control pattern of Figure 7g. Here, two segments are abutting orthogonally to the terminal segments of a Zöllner stack. The two abutting segments are collinear. There can be no Poggendorff effect, because the abutting angle is 90°. A Zöllner illusion due to a rotation of the stacks, or due to an orthogonal expansion of the stacks should produce no disruption of collinearity, but a shear deformation would produce a substantial disruption of collinearity—which is not observed. Actually, I carried out a very substantial orientation profile studies on the patterns of Figures 7f,g and their mirror-image patterns, with controls on pure misalignment effects. The results of this study run against the shear deformation explanation of the Zöllner illusion, and are in full support of the orthogonal expansion theory. It would even seem, from an illusory figure by Botti (1909), that orthogonal expansion might work better with oblique than with straight stacks Figure 7d).

In an exhaustive survey of tilt illusions, involving both known and new effects (Ninio and Pinna, 2006) we showed that a large number of variants of the Zöllner illusion could be described with a well-known metric effect, that of expansion of subdivided extents (the known prototype being Helmholtz square), and the adjunction of a simple clause: that the expansion effect occurs orthogonally to the dividing lines. Careful examination of Zöllner stacks indicate that this cannot be the whole story, since there are also effects on the lengths of the segments composing the stacks. Not too surprisingly, some articles in the field are misguided. The emphasis of Dakin et al. (1999) on the interaction between the orientation of the segments in the stack, and their envelope in the stack is misplaced, because the Zöllner effect can be shown to work with envelopes oriented in any direction.

Psychophysical work by Morgan on the Poggendorff illusion (Morgan, 1999) clearly indicates the importance of the local contacts between the parallels and the abutting segments. On the other hand, the illusory effects in the Zöllner family do not seem to require local contacts (see Figure 4d). So, there is, in my opinion, a dividing line between the two families of illusions.

### Trapezium, square-diamond and other illusions

I determined orientation profiles for two classical illusions, the trapezium illusion and 3 variants, and the square-diamond illusion and 7 variants (Ninio, 2011b). The trapezium illusion was maximal when the bases of the trapeziums were horizontal, and minimal when they were vertical. The oblique sides, but not the bases, were essential to the illusion, suggesting the existence of a common component between the trapezium and the Zöllner illusion. The study is made somewhat difficult by the fact that figures with trapeziums often lead to interpretations in perspective that perturb the comparison of trapeziums as flat figures (see also the paradoxical appearance in Figure 6j). One philosophically important side-result of the study is that two trapeziums in the standard configuration can never be altered in such a way as to be seen equal! When you try to equalize (by a nulling procedure) the two large bases and the orientations of the two sides, the small bases look unequal, and when you try equalize the two small bases and the orientations of the sides, then the two large bases look unequal. It is thus impossible to draw two trapeziums, one above the other, so that they would look identical.

The square-diamond illusion is usually presented with one apex of the diamond pointing toward the square (Figure 8f). I found that when the figures were displayed more symmetrically (Figure 8g) the illusion was significantly reduced. Furthermore, it is surpassed, for all subjects, by an illusion that goes in the opposite direction, in which the diagonal of a small diamond is underestimated with respect to the side of a larger square (Figure 6c and Ninio, 2011b).

## Discussion

My main motivation here was not to propose a theory on the origin of geometrical visual illusions (although I have one, see below) or discuss alternatives to the many circulating theories, that involve depth processing, eye movements, filtering, neuronal mechanisms, natural scene statistics and so on. I have been aiming at finding the most adequate formal description for what there is in an illusion. Typically, when we look at the Zöllner illusion, as usually presented (Figure 1m), in which the stack labeled L is a mirror-image of the stack labeled R, we may think that the stacks of opposite polarities L and R repel each other at one end, and attract each other at the other end. Experimental studies demonstrate the falsehood of this intuition, because whatever distortion we find in the usual displays with multiple parallel stacks of opposite polarities is also well detected with stacks of a single polarity. So, what happens to the Zöllner stacks? Intuitively again, we might describe the distortion as a (clockwise or counter-clockwise) rotation of the stack. Then, we might invoke physiological interpretations, involving orientation detectors in the visual cortex, and interactions between classes of orientation detectors that capture various aspects of a Zöllner stack. It is not my purpose to challenge or to support the physiological interpretations. I remain at a descriptive level, in a way reminding the early formal geneticists who described the laws of genetic transmission, before knowing the nature of the genetic material. So, my question is: does the distortion in the Zöllner stack amount to a rotation? There is at least one good argument to oppose to the rotation hypothesis of the stacks: the oblique segments that compose the stack are never perceived with an incorrect orientation. For instance, in Figure 7e, the verticality of the segments is clearly perceived, while at the same time, there is clearly an illusion. When we look at Figure 7e, another descriptive hypothesis comes to mind: that the segments composing the Zöllner stack slide with respect to their neighbors. Thus, in Figure 7e, it is as though the segments that are composing the stacks are sliding down differentially. In this case, the best description for the distortion in a Zöllner stack would be a shear deformation. However, a shear deformation would predict a misalignment of the small sides of the corners at the ends of the Zöllner stack of Figure 7g, but there is strictly no illusion there. One possibility remained, that the segments composing the stack is subject to an expansion that is orthogonal to the segments of which it is composed (see Figure 7c). Through the accumulation of experimental results (in collaboration with Kevin O'Regan) then the examination of a very large number of illusory patterns that seemed related to the Zöllner illusion (in collaboration with Baingio Pinna), I favored an orthogonal expansion principle to describe the distortions in the Zöllner illusion. This principle can describe a number of visual illusions not usually associated with the Zöllner illusion. These include the Helmholtz square illusion and the illusions of the Ehrenstein and Orbison family: The principle predicts, as a side effect, a regression to right angles tendency—see Figure 7b, and from there, other illusions such as Hering's illusion (Figure 1o) and perhaps tilt effects (e.g., Figure 4c). I also aggregate the trapezium illusion with Zöllner (see Figure 4i) A complicating factor in the experiments is the fact that the lengths of the segments are most probably subject to small variations from one end of a Zöllner stack to the other end. This needs to be documented.

The illusions of the twisted cord family are often associated with the Zöllner illusion, but I will draw a frontier between the two. The twisted cord (Figure 2l) and the related Fraser illusions are usually presented with at least three levels of gray. But there are weaker versions of these illusions in pure black outlines (Stuart and Day, 1988). The illusion strength has been studied as a function of the angle of the obliques composing a stack and the axis of the stack (e.g., Oyama, 1975), showing an inversion around 10–15° from a twisted cord to a Zöllner effect. This taken alone does not disprove the existence of a link between the two illusions. But there are also quite different effects associated with the two—it is not merely a question of sign reversal. In Figure 4g, I show a Zöllner effect with all-black or all-white stacks, and show the opposite effect with exactly the same geometry, but with an alternation of black and white sections. In Figure 2k a Münsterberg pattern is annotated. The A–B obliques would generate a Zöllner pattern, but the configuration with A and C well separated on the opposite sides of a segment seems to be essential for most of the illusions of the Münsterberg and Fraser family—see for the first the checkered patterns in (Wade, 1982); Wade, and for the second the variations by Kitaoka (1998) or Kitaoka et al. (2001).

Contrary to the pessimistic introduction to this review, I believe that some definite progress has been achieved in this area of “orientation illusions.” There are two main classes: an orthogonal expansion class, and a twisted cord class. The Poggendorff illusion is left out of this classification. It is, I believe, correctly described by a misangulation distortion (see Section Poggendorff), for which Morgan (1999) proposed a physiological explanation.

A large number of other illusions were discussed within a measurement framework. Assuming that the brain is constructing a representation from measurements taken on the illusory pattern, I proposed that a “convexity” principle (Section A Basic Metric Rule: The Convexity Principle) could well describe a number of effects. There is a difference between the traditional description by “contrast,” and the description by “convexity,” although both assume a perceptual increase of large over small extents. In the explanations involving contrast, two adjoining figures (for instance, a peripheral and a central circle in the illusion of Ebbinghaus) are compared. However, in many illusions, for instance the Müller-Lyer patterns of Figures 1a,b two equal segments seem to have different lengths, and a principle of assimilation with neighboring segments is then invoked. On the other hand, in the explanations involving convexity, measurements are taken over all the segments in a figure, a global representation is constructed, and compromises are made to deal with the discrepancies. Convexity alone can describe pure contrast effects (such as in Figures 5d,e,f) and, when complemented with the compromise principle, convexity can describe many effects usually described by an assimilation principle. Therefore, we have unified into a coherent framework two classes of explanations that seemed to be antagonistic. Here also, I think, there is a clear progress.

Yet, there are still many complications to deal with. It is as though the brain was using several instruments to construct a representation: a metric instrument to measure extents, an instrument to evaluate orientations (presumably, the edge detectors of the visual cortex), and perhaps instruments to measure collinearity, angles or curvature. Important differences in the magnitudes of the illusions in very similar patterns may arise from the fact that one pattern is interpreted with the help of a subset of the instruments whereas the geometry of the other pattern makes possible the use of an instrument that is outside the subset, and acts as a control device that counteracts the illusion.

One thing that strikes me, in relation to measurement theories is that we have several ways of capturing metric information through the eyes. We may view a scene by fixating a region with still eyes, we thus get a kind of camera picture that follows the rules of linear perspective. Or we may move our eyes over the scene in which case we derive metric information that obeys the rules of curvilinear perspective. However, we have a representation that does not seem to change as we move our eyes, then stop, then move them again. There is also the related isuue of shape constancy when the head or the body are tilted (e.g., Rock, 1990; Daini et al., 2003). We are also reminded that the results of psychophysical experiments—at least in the case of studies on the Poggendorff illusion—may reflect different types of processing, that vary with some conditions of stimuli presentation, as discussed by Gallace et al. (2012).

My conjecture is that evolution has devised a way to make us see a scene in an extremely stable fashion. To achieve this goal, the measurements taken with still eyes, and those taken with moving eyes are subject to distortions so as to make them agree (Ninio, 1998). We may also wonder whether or not the illusory effects are the same, when they are seen through the left, or the right eye? It is known that through stereoscopic vision, the brain can match the information flows from the two eyes with extraordinary precision. In particular, it matches the left and right projections of an oriented segment to deduce its orientation in 3d. From this, we may anticipate that visual illusions have the same magnitude whether measured through the left, or through the right eye, and perhaps conjecture that left and right projections are subject to transformations, prior to the operations of stereopsis, to bring them into comparable formats. By these arguments, I tend to believe that at least some geometric distortions that manifest themselves in visual illusions are deliberate, and have the goal to bring the visual representations into a single format, at least for the purpose of shape analysis, and make us see stable shapes, that do not vary with eye motion or the closing of one eye. Note that there are important differences in the viewing traditions of various populations, e.g., between a tribe of hunters, and a flock of television watchers.

It has been argued that our visual representation of shape is perhaps different from the representation that would guide motor responses, such as grasping an object or pointing at a target (e.g., Goodale and Milner, 1992 claimed that the preparation of the hand for grasping object was not influenced by illusory distortions; on the other hand, Melmoth et al., 2009 showed that pointing errors were subject to the Poggendorff distortions). I have no difficulty in accepting the idea that there may be several representations of shapes, and that these representations are tapped by different systems. It is clear, from visual memory work, that there are several representations of a same stimulus. As we go from iconic memory to short term visual memory then to deeper layers, the representations become more and more abstract. Yet, an abstract representation may work by its ability to activate a less abstract one (for instance, the word “circle” is a compact, abstract way of encoding the visual shape of a circle). It may well be that an early, topographic representation is used to direct hand movement rapidly, and that a more stable representation is constructed afterwards for a finer understanding of shape.

### Conflict of interest statement

The author declares that the research was conducted in the absence of any commercial or financial relationships that could be construed as a potential conflict of interest.
